# Enhancing the Role of Community Pharmacists in Medication Safety: A Qualitative Study of Voices from the Frontline

**DOI:** 10.3390/pharmacy13040094

**Published:** 2025-07-09

**Authors:** Annesha White, Erika L. Thompson, Solyi Kim, Janice A. Osei, Kimberly G. Fulda, Yan Xiao

**Affiliations:** 1 College of Pharmacy, University of North Texas Health Science Center, 3500 Camp Bowie Blvd, Fort Worth, TX 76107, USA; solyikim@my.unthsc.edu; 2 Department of Quantitative and Qualitative Health Sciences, The University of Texas Health Science Center at San Antonio, 7703 Floyd Curl Dr, San Antonio, TX 78229, USA; thompsone1@uthscsa.edu; 3 College of Pharmacy, University of Michigan, 428 Church Street, Ann Arbor, MI 48109, USA; j_osei@outlook.com; 4 Texas College of Osteopathic Medicine, University of North Texas Health Science Center, 3500 Camp Bowie Blvd, Fort Worth, TX 76107, USA; kimberly.fulda@unthsc.edu; 5 College of Nursing and Health Innovation, University of Texas at Arlington, 701 S Nedderman Drive, Arlington, TX 76019, USA; yan.xiao@uta.edu

**Keywords:** community pharmacists, medication safety, patient engagement, adverse drug events, qualitative research, medication therapy management (MTM)

## Abstract

Preventable adverse drug events (ADEs) remain a significant threat in community settings, a challenge that is critical in community pharmacy settings where continuity of care and healthcare access can be limited. This qualitative study explored the perspectives of 13 community pharmacists through focus groups and interviews to identify barriers and propose solutions to enhance their role in patient care. Pharmacists emphasized their critical position in ensuring safe medication use, particularly for older adults managing multiple chronic conditions. Key findings revealed five themes: (1) defining medication safety as minimizing risk and empowering patients; (2) characteristics of the “perfect patient,” including medication awareness and proactive engagement; (3) the pharmacist’s role in detecting and resolving medication-related problems; (4) systemic barriers such as time constraints, lack of access to patient records, insufficient privacy, and undervaluation of pharmacists’ roles; and (5) proposed solutions including private counseling areas, increased staffing, integrated electronic health records, and legislative recognition of pharmacists as healthcare providers. Strengthening collaboration with physicians and empowering patients through education were also highlighted as key strategies. These findings underscore the need for systemic changes—especially in light of lessons learned during the pandemic—to support pharmacists in delivering comprehensive medication management and improving patient safety.

## 1. Introduction

Medication safety is a cornerstone of effective healthcare delivery, requiring multiple layers of protection and collaboration among healthcare professionals and patients. Ensuring the safe use of medications is essential to minimizing the risk of adverse drug events (ADEs) and promoting positive health outcomes. In community settings, preventable medication-related harm remains a leading threat to patient safety, contributing to an estimated 4.5 million ambulatory care visits annually in the United States [1]. Addressing these harms is especially critical given that patients often receive care from multiple prescribers across various levels of care, increasing the risk of communication breakdowns and medication discrepancies.

A medication error is defined as any preventable event that may cause or lead to inappropriate medication use or patient harm while the medication is in the control of a healthcare professional, patient, or consumer [2]. While ADEs refer specifically to medication-induced harm, medication errors encompass a broader range of preventable incidents that can occur during prescribing, dispensing, administration, or monitoring. Patients and their families play a vital role in medication safety, particularly in ambulatory settings [3,4,5]. For example, insulin-related emergency department visits are often linked to patient self-management errors [6], and benzodiazepine-related ADEs are frequently associated with nonmedical use and self-harm [7]. Community pharmacists, as some of the most accessible healthcare professionals, are uniquely positioned to support patients and families in the safe use of medications. Studies show that patients visit community pharmacies nearly twice as often as they see their primary care physicians, underscoring the opportunity for pharmacists to intervene [8,9].

Despite this potential, numerous hazards to medication safety persist. An estimated 51 million dispensing errors occur annually in the U.S., often involving incorrect medications, dosages, or instructions [10]. Studies have also documented high rates of medication discrepancies during hospital discharge and transitions to community care, frequently due to incomplete medication histories or reconciliation errors [11,12]. These discrepancies contribute to preventable harm and are a major factor in hospital readmissions, with drug-related problems accounting for a median of 21% of readmissions—69% of which are considered preventable [13]. Approximately 60% of all medication errors occur during care transitions, often due to poor communication between healthcare providers, including between pharmacists and primary care physicians (PCPs) [14]. In particular, communication and collaboration between community pharmacists and primary care physicians remain underdeveloped, often hindered by a lack of interprofessional trust, limited access to shared patient records, and fragmented communication systems. These systemic barriers compromise the continuity of care and limit pharmacists’ ability to intervene effectively in medication management.

Improving medication safety in community settings requires a multifaceted approach that includes enhancing communication, strengthening medication reconciliation processes, and fostering collaborative medication management. Community pharmacists, as the final point of contact in the medication use process, are well-positioned to lead these efforts.

Pharmacists’ roles in medication safety are particularly critical during care transitions and in outpatient settings. With their expertise in pharmacotherapy, pharmacists can identify and resolve medication-related problems, promote adherence, and optimize therapeutic outcomes for both prescription and over-the-counter medications [15,16]. Enhanced pharmacist roles have been shown to reduce medication errors and hospital readmissions. A meta-analysis of 13 studies found that pharmacist-led interventions during care transitions resulted in a 37% reduction in medication errors and readmissions [17]. Similarly, a systematic review demonstrated that community pharmacists improve outcomes for chronic disease management through patient education and consultation [18].

However, despite this evidence, findings from a narrative review showed that clear roles of community pharmacists have not been well-accepted and supported across healthcare settings [15]. Moreover, there is limited research capturing the perspectives of frontline community pharmacists on their roles, challenges, and opportunities in promoting medication safety. Understanding these perspectives is essential not only for designing effective, context-sensitive interventions, but also for advocating for expanded pharmacy services and fostering interprofessional collaboration.

Community pharmacists are among the most accessible healthcare providers and often serve as the first point of contact for medication-related concerns. Understanding how pharmacists perceive and adapt their roles in response to evolving patient needs is vital for informing future pharmacy education, workforce preparedness, and policy development.

This study aims to fill these gaps by exploring the experiences of community pharmacists during the pandemic, identifying key barriers to medication safety, and highlighting strategies to empower pharmacists in enhancing patient care.

## 2. Materials and Methods

### 2.1. Sample and Recruitment

This qualitative study targeted licensed community pharmacists in the United States who provided care to older adults. Pharmacists were eligible if they served patients aged 65 and older who were managing five or more chronic medications—a population at elevated risk for medication-related harm due to polypharmacy, complex treatment regimens, and frequent care transitions. Eligibility was determined based on pharmacists’ self-report during recruitment. Pharmacists confirmed that they routinely provided care to older adults meeting the age and polypharmacy criteria as part of their daily practice. This information was verified through initial screening questions and reaffirmed during the opening portion of each interview or focus group. No external data systems (e.g., prescription databases) were used to verify patient demographics. Community pharmacists working with this demographic are uniquely positioned to identify safety risks and implement interventions, making their perspectives particularly valuable for informing medication safety strategies.

Participants were recruited through the professional networks of the research team, with additional participants identified via snowball sampling. Of the 13 participants, 8 were recruited through the research team’s professional networks, while 5 were identified through snowball sampling and outreach to the broader pharmacist community. To ensure a diverse representation of practice environments, email invitations were distributed to pharmacists working in chain, grocery store, and independent community pharmacy settings.

The final convenience sample consisted of 13 pharmacists practicing in Texas. All participants were actively engaged in community pharmacy practice and served older adults managing multiple chronic medications.

### 2.2. Data Collection

Data were collected through two virtual focus groups (with five and four participants, respectively) and four individual interviews, all conducted in September 2020. The use of both focus groups and individual interviews allowed for flexibility in scheduling and ensured broad participation. Participants were given the option to choose between a focus group or an individual interview based on their availability and comfort level. This approach was intended to maximize participation and accommodate varying schedules. While this method may introduce some self-selection bias, such as more outspoken individuals opting for focus groups, it also allowed for richer, more inclusive data collection by respecting participant preferences. Focus groups facilitated interactive discussions and peer exchange, while individual interviews enabled deeper exploration of personal experiences, particularly for those unable to attend group sessions. This mixed approach enriched the data and captured a wide range of insights.

We acknowledge the challenge of balancing breadth and depth in qualitative research. To address this, the interview guide was developed and refined by a multidisciplinary team with expertise in pharmacy, patient safety, and qualitative methods. Although a formal pilot study was not conducted, the first focus group served as an informal check to ensure that the questions elicited rich and meaningful responses. The combination of focus groups and individual interviews allowed for both breadth of perspectives and depth of individual experiences. Thematic saturation was achieved, indicating that the data collected were sufficient to capture the key themes relevant to the study objectives.

Eligible participants were licensed pharmacists, English-speaking, and able to participate virtually. All participants provided written informed consent and received a USD 50 gift card upon completing the session. The study protocol was approved by the University of Texas Arlington and the North Texas Regional Institutional Review Boards (Protocol #2019-163).

The sample size of 13 was guided by qualitative research principles that prioritize depth of understanding over generalizability. Recruitment continued until thematic saturation was reached, defined as the point at which no new themes or insights emerged from additional interviews. The consistency of responses across both focus groups and interviews indicated a high degree of thematic homogeneity, suggesting that further data collection was unlikely to yield substantially new information. This approach aligns with established qualitative research standards that support smaller sample sizes when saturation is achieved, and participants share relevant contextual similarities.

### 2.3. Procedures

Focus group and individual interview sessions took place virtually via Zoom for 90 min. Two researchers were present in each session: one as the moderator (non-pharmacist researcher with experience in qualitative data collection) and the other as a notetaker/observer. After every session, the two researchers reflected on the main areas covered during the session. The semi-structured focus group guide was developed with insight from researchers with a background in patient safety, pharmacy, and public health. Although the guide included a broad set of questions, moderators used a flexible approach, allowing the conversation to flow naturally, and prioritizing depth over breadth. Not all questions were asked in every session, and participants often addressed multiple themes within a single response. Focus group questions were discussed on the following topics: definition of medication safety, top medication-related harms at home, barriers to preventing medication-related harms at home, what a perfect patient would look like, the role of pharmacists in medication safety, factors that discourage collaboration between pharmacists and primary care providers (PCPs), and strategies for medication safety and/or collaboration with PCPs (Table 1). Immediately following the focus group, the video portion was destroyed to protect confidentiality, and the focus group audio was transcribed verbatim using a professional transcription agency.

Although a formal pilot study was not conducted, the interview guide was developed and refined by a multidisciplinary team with expertise in pharmacy, patient safety, and qualitative research. The guide was reviewed for clarity and relevance, and the first focus group served as an informal check to confirm that the questions elicited meaningful and contextually appropriate responses from participants.

### 2.4. Data Analysis

Thematic analysis was conducted using a deductive coding approach based on the interview guide. The analysis was informed by a constructivist paradigm, which assumes that knowledge and meaning are co-constructed through participants’ experiences and interactions [19]. This perspective guided our interpretation of the data, allowing us to explore how community pharmacists make sense of their roles, challenges, and opportunities in promoting medication safety. Verbatim transcripts from the focus groups and interviews were imported into Microsoft Excel for coding. Two researchers independently reviewed and coded the transcripts to ensure consistency and reduce individual bias. The initial coding scheme was developed from the interview guide and included predefined categories such as definitions of medication safety, barriers to safe medication use, pharmacist roles, patient behaviors, and proposed solutions. These primary codes were refined iteratively through team discussions as new insights emerged.

To ensure coding reliability, the two coders compared their results and resolved discrepancies through consensus discussions. A third team member was available to adjudicate disagreements, though this was rarely necessary. Inter-coder agreement was assessed informally during the reconciliation process, and coding consistency was monitored throughout the analysis. Data saturation was achieved when no new themes or subthemes emerged from the final interviews. This was determined through ongoing review of the transcripts and coding summaries, with the research team noting redundancy in participant responses and thematic content.

To ensure data quality, the research team conducted regular debriefing sessions after each interview and coding round to check for completeness, consistency, and alignment with the study objectives. Transcripts were reviewed for accuracy against the audio recordings prior to analysis. Although Microsoft Excel was used for coding and organizing data, no specialized qualitative analysis software (e.g., NVivo or ATLAS.ti) was employed. The team opted for Excel due to the manageable dataset size and the structured nature of the coding framework.

## 3. Results

### 3.1. Participant Characteristics

The two focus groups and individual interview sessions included a diverse group of participants from various pharmacy-related roles and companies. Specifically, there were five pharmacy managers, one pharmacy supervisor, and seven staff pharmacists (Table 2). These individuals represented major pharmacy chains and independent pharmacy. This mix of participants provided a broad perspective on the issues discussed, as they brought insights from different levels of pharmacy operations and from various organizational cultures. The inclusion of both managers and frontline pharmacists ensured that the discussions covered a wide range of experiences and viewpoints, from strategic oversight to day-to-day patient interactions. This diversity in roles and affiliations helped to enrich the conversation and provided a comprehensive understanding of the challenges and opportunities within the pharmacy sector.

### 3.2. Defining Medication Safety

The study identified five key themes from pharmacist interviews, highlighting their perspectives on medication safety and the challenges they face (Table 3). One major theme was defining medication safety, where pharmacists emphasized minimizing risks and empowering patients with medication knowledge. As one pharmacist explained, “I think there’s kind of two perspectives for medication safety. Pharmacists’ perspective is the right drug, right directions, right patients with some comprehensive counseling attached to it. I think from a patient’s perspective; it’s really just empowering them with knowledge about their medication (FG 01, P2, Pharmacy Manager).”

### 3.3. Characteristics of the “Perfect Patient”

In addition, the perfect patient was identified as an individual who is aware of their medications, brings a list to share with pharmacists and doctors, adheres to their medication regimen, and is prepared with questions for the pharmacist. Pharmacists play a crucial role in medication safety through the provision of extensive drug utilization reviews. However, a common misconception exists—as one participant noted, “I think a big one is that they [patients] feel like they’re bothering us or annoying us with all their questions. Or if they’re a little confused about something, they might be embarrassed to ask (FG 02, P7, Staff Pharmacist).”

### 3.4. Pharmacists’ Roles in Medication Safety

In contrast, the pharmacists provided feedback on some of the most common barriers that they face in the community setting. Pharmacists face barriers to counseling such as time constraints, lack of access to patient records, an undervalued role in healthcare, and insufficient privacy in pharmacies. One pharmacist illustrated the critical nature of their counseling role through a powerful example involving a patient who had been without his anticoagulant medication for over two weeks due to cost concerns. The pharmacist recalled the following: “I told him, I said, look, this decision right now could be a matter of life and death. There’s no way for you to know how quickly you could potentially clot and have a stroke. If I were your daughter or your wife, I would not want you to take that chance (ID 02, P11, Pharmacy Manager).” This moment underscores the pharmacist’s role not only in medication dispensing, but also in urgent, empathetic decision-making that directly impacts patient safety.

Another pharmacist noted “Time is a huge, you know, limiting factor that we have in order to be the ideal pharmacist (FG 01, P1, Pharmacy Supervisor).” To enhance medication safety, pharmacists also suggested solutions which included creation of separate counseling spaces for private consultation areas, increasing the number of pharmacists and support staff to provide adequate time for counseling, raising awareness and education to increase legislative recognition of the pharmacist’s role, and better integration of electronic communication systems.

### 3.5. Barriers to Medication Safety in Community Pharmacy

Community pharmacists encounter several challenges that impact their ability to ensure medication safety. Table 4 highlights these barriers, including time constraints, an undervalued role in patient care, lack of access to patient files, insufficient privacy for consultations, limited integration into the healthcare community, and communication issues with other healthcare providers.

Time constraints remain one of the most significant barriers, as pharmacists often have limited opportunities for comprehensive patient counseling and medication reviews due to high workloads and busy pharmacy environments. One pharmacist stated “So the greatest limitation we have in retail community pharmacies is that there is not enough time in an ideal world to really do some of this as we are saying because, you know, you are, you are so busy… (FG 01, P4, Pharmacy Manager)” Additionally, pharmacists shared that their roles are frequently undervalued within the healthcare system: “They [patients] don’t have a good understanding of what, you know, we can do or what we do back there in a pharmacy (FG 02, P8, Staff Pharmacist).” “Patients treat the pharmacy like, you know, like it was a store. You know, you go in, you get to the register, and you check out really quick, fast, easy, not realizing that it’s still a bunch of steps that go behind there (FG 01, P4, Pharmacy Manager).”

A lack of access to patient files further complicates pharmacists’ efforts to ensure medication safety. As one pharmacist stated, “in an ideal, perfect world, all doctors, all pharmacists, we all had access to it [the patient’s health information], and we could see everything in one place and communicate through that as well (FG 02, P7, Staff Pharmacist).” Another significant issue is the lack of privacy within community pharmacies. Many pharmacy layouts do not provide dedicated spaces for private consultations. “The [pharmacy] counter is not useful because the patient, sometimes the patient, can’t even see us behind the counter (FG 02, P8, Staff Pharmacist).” Furthermore, “pharmacies are just too open to the public, and everyone can hear every conversation going on (FG 02, P7, Staff Pharmacist).” Additionally, community pharmacists often struggle with limited integration into the broader healthcare system. “They [physicians] should trust pharmacists that they know more about the medications and what is going on (FG 01, P5, Pharmacy Manager).” Communication issues with PCPs further compound this problem. As one pharmacist states, “Doctors are extremely hard to get a hold of… (FG 01, P2, Pharmacy Manager)”; moreover, they stated that “I think sometimes the problem is that our concerns get stuck with the medical assistant and never reach the physician (FG 01, P3, Staff Pharmacist).”

Additionally, pharmacists shared their perspectives on the characteristics of an ideal physician collaborator and the misconceptions that hinder effective teamwork. These insights, captured during focus group discussions and reflected in Table 1, underscore the importance of mutual respect, open communication, and shared responsibility in optimizing patient care.

### 3.6. Strategies and Solutions for Enhancing Medication Safety

To address these challenges, several solutions have been proposed to enhance pharmacists’ ability to contribute to medication safety (Table 4). One key strategy is the establishment of dedicated counseling spaces within pharmacies. By providing private areas for patient consultations, pharmacists can foster more meaningful interactions, encourage open discussions about medication concerns, and ensure confidentiality in patient care. “… [pharmacists and patients] need the separate little room, more of a clinic-type setting in the pharmacy than just being open with chairs (FG 02, P7, Staff Pharmacist).” Increasing the number of pharmacists and support staff is another crucial solution that was suggested. Pharmacists stated that “What we need is more staff in pharmacy so we can set aside time to talk with, sit, and talk with patients (FG 02, P7, Staff Pharmacist).” With additional personnel, pharmacists can allocate more time to patient care, conduct thorough medication reviews, and focus on patient education without being overwhelmed by dispensing duties.

Legislative changes that formally recognize and support the expanded roles of pharmacists are another essential solution towards improving medication safety. Enacting policies that acknowledge pharmacists as integral members of the healthcare team can help elevate their status and expand their responsibilities in patient care. One pharmacist stated “We’re not really recognized as a healthcare provider. So a lot of doctors may not think of us as part of the healthcare team and that we should collaborate together. They kind of just think of us as the pill dispensers, pouring pills in a bottle (FG 02, P7, Staff Pharmacist).” Another pharmacist mentioned “So I think that’s definitely a big barrier is that I feel like us as pharmacists, I wish we could really come together and show other healthcare professionals, providers, what do we do. We can be a huge source of information if you need us to find something for you in relation to a medication, whether it’s a drug–drug interaction, dosing, contraindications, or monitoring, we can help (ID 04, P13, Staff Pharmacist).” Lastly, implementing integrated electronic communication systems was identified as a vital step in enhancing pharmacists’ contributions to medication safety. Strengthening collaboration with PCPs through technology, such as a universal electronic medical record (EMR), could improve communication and patient health outcomes. As one pharmacist stated, “… in an ideal, perfect world, all doctors, all pharmacists, we all had access to it [the EMR], and we could see everything in one place and communicate through that as well (FG 02, P7, Staff Pharmacist).”

## 4. Discussion

Several studies have demonstrated the impact of community pharmacists in improving medication safety [17,20,21]. One such initiative in the literature confirms our findings on the positive impact that community pharmacists have on improving safety. The study involved a team of community pharmacists conducting medication safety reviews for Medicare Part D beneficiaries, identifying 36,455 medication-related problems across nearly 23,000 beneficiaries over two years [20]. Moreover, an economic evaluation of pharmacist-led interventions highlighted that those patients who received a pharmacist-led medication review experienced a 0.6% reduction in total medical expenditures, whereas those who did not engage with pharmacists saw a 15.5% increase in costs [21]. Most notably, the pharmacist-driven medication safety reviews were associated with a 3.8% reduction in mortality rates, emphasizing the significant role pharmacists play in optimizing patient outcomes [21].

In addition to earlier findings, recent studies further underscore the expanding role of pharmacists in preventing medication errors and improving patient outcomes. For example, pharmacist-led interventions have been associated with reductions in adverse drug events, improved medication reconciliation, and enhanced patient safety during transitions of care [22,23,24]. These findings reinforce the relevance of our study and highlight the global momentum toward integrating pharmacists more fully into patient care teams

This study was conducted in Texas, a state where pharmacists are permitted to engage in collaborative practice agreements (CPAs) with physicians. Under these agreements, pharmacists may initiate, modify, or discontinue drug therapy in accordance with a physician-approved protocol. While pharmacists in the U.S. do not have independent prescribing authority in most states, CPAs allow for expanded clinical roles in medication management. Most outpatient prescriptions in the U.S. are written by primary care providers, with hospitals primarily responsible for inpatient prescribing. These structural elements shape the opportunities and limitations community pharmacists face in contributing to medication safety. Although the findings are grounded in the Texas context, the themes, such as the need for better integration, communication, and recognition of pharmacists’ roles, are relevant to international audiences. For non-U.S. healthcare managers and pharmacists, the study highlights the importance of enabling pharmacist-provider collaboration, ensuring access to patient information, and addressing systemic barriers that limit pharmacists’ clinical contributions.

Regarding medication safety reviews, a 2024 report revealed over two million cases that were reported to the FDA Adverse Events Reporting System (FAERS) [25]. Among these, approximately one million cases involved serious adverse drug events (ADEs) that were either life-threatening or required hospitalization, while 148,463 cases resulted in death [25]. The high incidence of ADEs underscores the urgent need to enhance medication safety measures to minimize patient harm and improve overall health outcomes. Studies show that community pharmacists, with their specialized expertise in medication management, are in a unique position to mitigate these risks [18,26].

While the opportunity exists for community pharmacists to lead the way in improving medication safety, several obstacles hinder their ability to maximize their contributions to medication safety. This study highlights those barriers and confirms the related literature. For example, some qualitative analyses have shown that one of the most pressing challenges is the lack of interprofessional trust and respect among healthcare providers [26,27]. Many prescribers continue to view pharmacists primarily as medication dispensers rather than as clinical experts capable of preventing medication errors. This misconception limits pharmacists’ ability to intervene in medication management, resulting in missed opportunities to enhance patient safety. Another significant barrier noted in our findings emphasizes the impeding impact of the community pharmacist’s restricted access to patient medical records, including lab results, diagnoses, and medication histories. Studies show that without comprehensive patient information, pharmacists struggle to perform thorough medication reviews and address potential drug-related issues effectively [28,29].

To overcome these challenges, the solutions suggested by community pharmacists participating in this study focus on data integration and collaboration. A prospective study supports the benefits of implementing an electronic health information system that provides community pharmacists with real-time access to patient medical records, including lab values, medication lists, and diagnostic information [30]. The HomeCoMe (Home-based Community Pharmacist-led Medication Management) program demonstrated the effectiveness of such integration [30]. The study highlighted that community pharmacists were able to identify and resolve 83.6% of drug-related problems post-hospital discharge, with the most common issues being the need for additional education (36.1%) and medication non-compliance (16.4%) [30]. Improved access to patient information would enable pharmacists to proactively identify medication discrepancies, prevent adverse drug interactions, and provide comprehensive medication management services.

To bridge these gaps, pharmacists in this study emphasized the importance of CPAs and integrated electronic health records (EHRs) as mechanisms to strengthen interprofessional collaboration. CPAs allow pharmacists to work directly with prescribers to initiate, modify, or discontinue medications based on patient needs, thereby formalizing their clinical role within the healthcare team [31]. Integrated EHR systems would provide pharmacists with real-time access to patient data, enabling more informed decision-making and reducing the risk of medication errors during care transitions. Additionally, fostering interdisciplinary collaboration through regular case discussions and joint medication reviews can further enhance communication and trust between pharmacists and primary care providers. Evidence suggests that such collaborative practices improve patient outcomes, reduce medication errors, and enhance overall healthcare efficiency [32]. Medication errors can also be minimized through collaborative efforts from patients. As highlighted in the study, patients who are well-informed about their medications, actively maintain and share an updated list of their prescriptions, adhere to their prescribed regimens, and demonstrate a willingness to learn more about their treatments are better equipped to prevent medication-related problems. By taking an active role in their own medication management, patients can enhance communication with healthcare providers, reduce the risk of errors, and ultimately improve their overall health outcomes [33,34].

Lastly, the community pharmacists in this study noted time constraints and limited access to private counseling spaces as barriers. Several recently published studies also report on these concerns, noting that heavy workloads and staffing shortages often prevent pharmacists from dedicating sufficient time to patient care, reducing opportunities for in-depth medication counseling and intervention [29,35,36]. Increasing the number of reliable pharmacy staff members can help distribute the workload more effectively, allowing pharmacists to focus on their clinical responsibilities. A retrospective study by Liaw and colleagues successfully demonstrated the impact of pharmacy technicians as additional staff members with benefits shown in their identification of medication discrepancies [11]. This study also noted the harmful effects resulting from the lack of designated private areas for consultations, such as patient discouragement from discussing sensitive medication-related concerns. Establishing dedicated counseling spaces within pharmacies can enhance patient privacy, encourage meaningful discussions, and ultimately improve medication adherence and patient health outcomes [27].

While many of the barriers and solutions identified in this study align with the existing literature, this research offers a unique contribution by capturing the lived experiences and nuanced perspectives of frontline community pharmacists in their own words. Unlike prior studies that often focus on quantitative outcomes or institutional-level interventions, this study provides rich, qualitative insights into how pharmacists conceptualize medication safety, navigate systemic constraints, and envision ideal patient and provider relationships. Notably, the theme of the “perfect patient”—defined by pharmacists as one who is informed, proactive, and communicative—emerged as a novel construct that has received limited attention in the pharmacy literature. This concept may serve as a foundation for future research exploring patient engagement strategies and pharmacist–patient communication models. Additionally, the study reinforces the importance of context-specific barriers, such as lack of privacy and workflow limitations, which remain under-addressed in policy and practice.

The findings also have important implications for pharmacy education and workforce development. The challenges and adaptive strategies described by pharmacists during the pandemic underscore the need to integrate real-world crisis preparedness, interprofessional communication, and patient engagement skills into pharmacy curricula. Educators should consider incorporating case-based learning and simulations that reflect pandemic-era scenarios to better prepare students for future public health emergencies. These educational enhancements can help ensure that future pharmacists are equipped not only to manage routine medication safety responsibilities but also to respond effectively during healthcare system disruptions.

### Limitations

While this study highlights the promising role of community pharmacists in enhancing medication safety, several limitations should be acknowledged. First, the findings are based on interviews with a small sample of 13 pharmacists, which may not fully capture the breadth of experiences and perspectives across different geographic regions or practice settings. Although thematic saturation was achieved, the limited sample size restricts the generalizability of the results.

Second, although the inclusion of pharmacists from various community pharmacy types (e.g., chain, grocery, and independent) and roles (e.g., staff pharmacists, managers, and supervisors) enriched the dataset, the study did not explore potential differences in perspectives based on these variables. Due to the small sample size, subgroup analysis was not feasible. Future research with a larger and more stratified sample could help uncover how role and setting influence pharmacists’ views and experiences related to medication safety.

Third, the study focused solely on the perspectives of pharmacists and did not include input from other healthcare providers such as primary care physicians, nurses, or patients. This limits the ability to fully understand interprofessional dynamics and barriers to collaboration. Future studies should incorporate a broader range of stakeholders to develop more comprehensive and actionable strategies for improving medication safety in community settings.

Fourth, all interviews and focus groups were conducted virtually, which may have influenced participant engagement and the depth of responses due to limitations in nonverbal communication and rapport-building. While virtual methods offered flexibility and safety during the study period, they may not fully replicate the dynamics of in-person interactions [37].

An additional limitation is the potential for selection bias due to the recruitment of participants through the research team’s professional networks. While this approach facilitated access to experienced community pharmacists, it may have introduced a degree of positive bias, as participants could have been more aligned with the interviewer’s perspectives or more willing to participate due to existing relationships. This possibility was considered during data interpretation, and efforts were made to ensure transparency and reflexivity throughout the research process

Finally, this study employed a qualitative design to capture community pharmacists’ unique perspectives. Although the goal was to provide some context for their insightful experiences and challenges, the absence of quantitative analysis limits the generalizability of the findings. Future research could use quantitative methods, such as structured surveys or mixed-methods designs, to validate and extend these findings across broader populations. Such approaches would allow for statistical comparisons and enhance external validity.

## 5. Conclusions

This study reinforces the essential role of community pharmacists in promoting medication safety, particularly for older adults managing complex medication regimens. Through qualitative insights from frontline pharmacists, the study highlights persistent barriers—such as time constraints, limited access to patient records, and lack of privacy—that hinder pharmacists’ ability to deliver optimal care. It also identifies practical, system-level solutions, including the need for integrated electronic health records, legislative recognition of pharmacists as healthcare providers, and improved collaboration with primary care physicians.

While many of the challenges and recommendations align with the existing literature, this study contributes a unique perspective by capturing pharmacists’ lived experiences and their conceptualization of the “perfect patient”—a proactive, informed individual who engages meaningfully in their own care. This novel theme offers a valuable foundation for future research on patient engagement and pharmacist–patient communication strategies.

To fully realize the potential of community pharmacists in enhancing medication safety, systemic changes are needed to support their clinical roles, improve interprofessional collaboration, and empower patients. By addressing these areas, healthcare systems can reduce preventable medication errors, improve care transitions, and advance patient-centered outcomes.

Future research should build on these findings by exploring the identified themes in greater depth and across broader populations. Mixed-methods designs, which combine the depth of qualitative inquiry with the generalizability of quantitative analysis, may be particularly well-suited to examine the prevalence and impact of barriers and solutions identified in this study. Additionally, longitudinal studies could assess how changes in policy, technology, or workflow affect pharmacists’ roles and patient outcomes over time. Such approaches will help generate actionable insights and inform the development of scalable interventions to strengthen the role of community pharmacists in medication safety.

## Figures and Tables

**Table 1:** Community pharmacist medication safety focus group questions and objectives.

Question Area	Focus Group/Individual Interview Questions	Purpose
Initial Thoughts and Background	Can you tell me a little about yourself, including your specialty, board certification, and number of years in practice since graduation?	Sets the stage, reveals initial perceptions, and allows for free expression.
Opinions and Perceptions	How would you define medication safety? Please share your views on patient medication safety in terms of outcomes and pharmacist activities related to medication safety, as well as medication related harms that concern you. Think especially about older adults with high-risk medications or multiple medications.	Gathers feedback on medication safety viewpoints.
Use and Experience	What were some of the indications that patients may be of high risk for medication related harms at home? In general, among your patients who are 65 years or older, what do you see as top medication-related harm? Are there classes of medications that tend to cause problems for patients? Do you see problems often associated with hospitalizations, such as medication reconciliation, lack of transition care, communication with patients or with you, medication labeling, or patient education? Do you see problems often caused by our healthcare systems, such as insurance and fragmented systems? Do you see problems often associated with patient factors, such as health literacy? In your pharmacy, are there other ways that you interact with patients (e.g., in person over the counter visits vs. drive through visits)? What misconceptions do you believe patients have about pharmacists?	Encourages detailed accounts and real-life scenarios related to use.
Solutions and Barriers	What did you do to reduce potential medication-related harms? What were the barriers if any for you to work with this patient to prevent medication related harm at home?	Identifies pain points and areas for improvement.
Solutions	What were some of the practical solutions you used to work with the patient to prevent medication related harm at home?	Explores solutions and highlights strengths.
Recommendations	If you could imagine your perfect patient, what would they look like (i.e., bring a medication list, prepared questions, brown bags, etc.)?	Uncovers different viewpoints and encourages discussion.
Use and Experience	Now let’s talk about the role of pharmacists in medication safety. Can you walk me through a memorable experience in which you helped, or tried to help, a patient reduces drug-related problems or risk? Have you ever had recent experiences in which you were very concerned about potential medication related harm?	Encourages detailed accounts and real-life scenarios related to use.
Recommendations	In an ideal world, what role should pharmacists play to reduce medication related harms? How do you see the role differences if any between an independent pharmacist and a chain pharmacist. Please share specific examples of barriers unique to independent versus chain pharmacists in reducing medication related harms.	Identifies factors that influence the pharmacists’ role.
Recommendations	What questions or phrases do you use to start conversations about safety concerns or issues with patients? Please share any experiences in which you have to explain your roles as a pharmacist. What would help you to play that ideal role?	Provides actionable advice and insights for those who are unsure.
Recommendations	Consider the location of your pharmacy, how many physicians do you know that are within a 10-mile radius? Please elaborate on your relationship with those physicians. Please share an example in which you worked with a family physician or primary care office to help a patient with medication safety? Tell me a story…	Provides actionable advice and insights and encourages detailed accounts and real-life scenarios related to use.
Recommendations	If you could imagine your perfect physician, what would they look like (i.e., encourage a medication list, refer patients for consultation, answer phone calls, respect my opinions, etc.)? What are the factors that discourage collaboration between pharmacists and family physicians? What are some of the misconceptions by primary care physicians that hurt the collaboration? What are some of the misconceptions that pharmacists tend to have of PCPs? Please reflect and share your thoughts on strategies for medication safety and/or collaboration with physicians. What solutions do you foresee in the near future that may be helpful to improve patient health outcomes?	Provides actionable advice and insights for those who are unsure.
—	Is there anything else you’d like to say about medication safety and the community pharmacist’s role?	Allows for open and spontaneous expression.

**Table 2:** Characteristics of community pharmacist participants by session type, pharmacy setting, and role.

Session Type	Participant Identification	Practice Setting	Pharmacist Role
Focus Group 1 (FG 01)	Pharmacist 1 (P1)	Chain Pharmacy	Pharmacy Supervisor
	Pharmacist 2 (P2)	Chain Pharmacy	Pharmacy Manager
	Pharmacist 3 (P3)	Chain Pharmacy	Staff Pharmacist
	Pharmacist 4 (P4)	Chain Pharmacy	Pharmacy Manager
	Pharmacist 5 (P5)	Grocery Store Pharmacy	Pharmacy Manager
Focus Group 2 (FG 02)	Pharmacist 6 (P6)	Chain Pharmacy	Pharmacy Manager
	Pharmacist 7 (P7)	Chain Pharmacy	Staff Pharmacist
	Pharmacist 8 (P8)	Chain Pharmacy	Staff Pharmacist
	Pharmacist 9 (P9)	Grocery Store Pharmacy	Staff Pharmacist
Individual Interview (ID 01)	Pharmacist 10 (P10)	Chain Pharmacy	Staff Pharmacist
Individual Interview (ID 02)	Pharmacist 11 (P11)	Independent Community Pharmacy	Pharmacy Manager
Individual Interview (ID 03)	Pharmacist 12 (P12)	Chain Pharmacy	Staff Pharmacist
Individual Interview (ID 04)	Pharmacist 13 (P13)	Chain Pharmacy	Staff Pharmacist

Legend: FG 01 = Focus Group 1; ID 01 = Individual Interview 1; P1 = Pharmacist 1.

**Table 3:** Key themes from community pharmacist focus group and interview sessions.

Themes	Descriptions
Define Medication Safety	Minimizing risk or free from harm; Patient empowerment to know about their medications; Patient health literacy; Patient counseling and asking patient questions about their medications; Specific areas of concern for older adults: opioids, sleep hygiene.
Perfect Patient	Aware of their medications (e.g., what they are for, when they take them); Bring a list of their medications to share with their pharmacist and doctor; Sharing a list of their medications prior to coming to the pharmacy; Adherence with medications; Prepared with a list of questions for the pharmacist; Wants to recover.
Role of the Pharmacist in Medication Safety	Problem detection (e.g., drug utilization review, brown bag review of medications); Barriers to detecting drug-related risks (e.g., time constraints and issues communicating with PCP); Type of problem (e.g., side effects, drug–drug interaction, adherence, duplications); Problem resolution (e.g., referral to PCP, medication change, creating a medication list, changing the medication schedule).
Barriers of Concern	Time; Undervalued role as pharmacists; Lack of access to patient files (e.g., diagnosis, labs); Lack of integration into the community to provide patient education; Open counseling area and lack of privacy.
Solutions for Improving Medication Safety	Separate space for counseling; Having more pharmacists/staff available; Legislation recognizing the role of pharmacists; Integrated system; Close collaboration between community pharmacist and PCP; Reassure PCP that pharmacists are part of the healthcare team; Relationship building; Technology to improve communication (e.g., universal electronic medical record).

**Table 4:** Barriers to medication safety faced by community pharmacists and solutions for improving community pharmacist roles.

Barriers	Solutions
Time Constraints	Increased Staffing
Lack of Privacy	Separate Spaces for Counseling
Undervalued Role	Legislation Recognizing Pharmacists’ Roles
Integration into the Community	
Lack of Access to Patient Files	Integrated Electronic Communication Systems
Communication Issues	

## Data Availability

The data presented in this study are available on request from the corresponding author. The data are not publicly available due to privacy or ethical restrictions.
